# Screening for pre-clinical disability in different residential settings

**DOI:** 10.1186/1471-2318-10-52

**Published:** 2010-08-03

**Authors:** Kate Gibson, Lesley Day, Keith D Hill, Damien Jolley, Stuart Newstead, Flavia Cicuttini, Leonie Segal, Leon Flicker

**Affiliations:** 1 Department of Epidemiology and Preventive Medicine, Monash University, Melbourne, Australia; 2 Monash University Accident Research Centre, Monash University, Melbourne, Australia; 3 School of Physiotherapy, Latrobe University, Melbourne, Australia; 4 Northern Health, Melbourne, Australia; 5 National Ageing Research Institute, Melbourne, Australia; 6 Division of Health Sciences, University of South Australia, Adelaide, Australia; 7 Western Australia Centre for Health and Ageing, University of Western Australia, Perth, Australia

## Abstract

**Background:**

Preventing disability and offering effective interventions to older people during early decline in function is most likely to be effective if those most at risk of progressive disablement are able to be identified. Similarly the ability to easily identify a group with similar functional profile from disparate sectors of the community is of significant benefit to researchers. This study aimed to (1) describe the use of a pre-clinical disability screening tool to select a functionally comparable group of older men and women with early functional limitation from different settings, and (2) explore factors associated with function and disability.

**Methods:**

Self-reported function and disability measured with the Late-Life Function and Disability Instrument along with a range of physical performance measurements were compared across residential settings and gender in a sample of 471 trial participants identified as pre-clinically disabled after being screened with the Fried pre-clinical disability tool. Factors that might lie on the pathway to progressive disablement were identified using multiple linear regression analysis.

**Results:**

We found that a sample population, screened for pre-clinical disability, had a functional status and disability profile reflecting early functional limitation, regardless of residential setting or gender. Statistical models identified a range of factors associated with function and disability which explained a greater degree of the variation in function, than disability.

**Conclusions:**

We selected a group of people with a comparable function and disability profile, consistent with the pre-clinical stage of disability, from a sample of older Australian men and women from different residential settings using the Fried pre-clinical disability screening tool. The results suggest that the screening tool can be used with greater confidence for research, clinical and population health purposes. Further research is required to examine the validity of the tool. These findings offer insight into the type of impairment factors characterising early functional loss that could be addressed through disability prevention initiatives.

**Trial Registration:**

ACTRN01206000431527

## Background

In common with many other countries, Australia has an aging population[1,2]. The proportion of people aged 65 years and over is projected to rise from 13% in 2002 to between 26% and 38% by 2051[2]. At the same time the rate of disability among older people in Australia, the United Kingdom and the Netherlands is not declining[3]. In Australia, there has been a consistent increase in the overall rate of disability for almost two decades[3,4]. Disease and disability are not an inevitable part of aging [5] and rates of disability are subject to improvements in technology, economic status and management [6]. The particular methods of disability measurement will influence the reporting of disability and thus our understanding of the patterns of disability. Nonetheless, it remains the case that independent older adults are at risk of developing dependence and disability as a result of progressive decline in function and mobility[7]. Preventing disability and offering effective interventions to older people during early decline in functional status is a growing public health imperative.

The pathway of progressive disablement is well described. Disability can be defined as the expression of a functional limitation in a social context[8]. Theoretical models describe disability as the interrelationship between activity limitations, impairments, and health status against a background of contextual factors[8,9]. Although the process is not always linear, the end result is a restriction or lack of ability to perform an activity within the normal range[10]. Pre-clinical disability refers to a transitional state on the disablement pathway between impairment and disability that is characterised by early changes to task performance, but precedes self perception of difficulty with task performance[11]. This may represent a self-reported state of frailty, before the cascade of events leading to subsequent disability[12]. Consequently, the ability to identify those people who are at high risk of disability although not yet disabled offers the opportunity to prevent the onset of disability[11].

Self-reported measures of physical disability assess difficulty, inability or degree of assistance required to perform specific tasks of mobility, household management or personal care[13]. However, because people with pre-clinical disability due to impairment are still able to accomplish a task under certain circumstances without perceiving a difficulty, the above measures may not be sensitive enough to recognise early functional decline[11,13]. Although research has shown that objective measures of physical performance, such as lower extremity muscle strength and walking speed, are good predictors of pre-clinical disability in older adults [14], self report measures are often the only feasible approach to identifying disability for both research or population health purposes. For this reason an effective self reported measure was developed by Fried and colleagues that identifies individuals in the pre-clinical stage of disability by report of modification of their method of performing mobility tasks which compensate for the impact of underlying health changes[7]. Fried and colleagues found self report of task modification and performance measures to be independent and strong predictors of incident mobility disability[7]. The test re-test reliability and predictive validity have been reported in studies of older American women [13], late middle aged African Americans [15], and older sedentary Finnish people [16]; all three studies involved community dwelling participants.

Despite the potential benefits of easily identifying a vulnerable subset of older people who are at increased risk of progression to mobility disability, there is currently limited information on the use of the Fried screening tool. The aims of this study were two-fold. First, to describe the use of the self reported pre-clinical disability screening tool in both retirement village dwelling and community dwelling older Australian men and women. In our sample, defined as pre-clinically disabled with the Fried screening tool, we expected to find (1) similar functional status and disability stage regardless of residential setting or gender, and (2) a functional and disability profile matching that expected for a pre-clinically disabled group. Performance measures and self reported disability were compared across residential settings and gender in order to describe the function and disability characteristics of our baseline sample group. Second, to explore factors associated with function and disability in a series of models.

## Methods

### Participants

**Figure 1:**
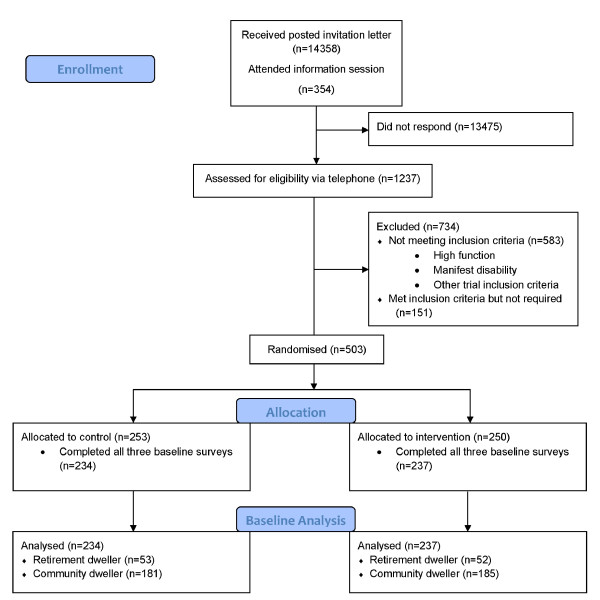
**Recruitment and randomisation of eligible participants, Exercise for Independent Living trial, Melbourne, Australia**.

Residents from retirement villages and private community dwellings in metropolitan Melbourne were included in this cross sectional study. This sample was drawn from that of a larger randomised control trial testing the efficacy of Tai Chi in delaying disability onset (Exercise for Independent Living trial- ExIL). Invitation letters were sent to 14,358 people aged 70 years and over registered on the Australian electoral roll living in selected retirement villages or close to the specific facilities for the intervention classes (Figure 1). Information sessions were held in local retirement villages. Interested potential participants, receiving a letter or attending a session, contacted the research team and were screened for eligibility by telephone. The Fried pre-clinical disability screening tool was administered via telephone to 1237 eligible volunteer respondents, from whom 654 were categorised as having pre-clinical disability in addition to meeting all other eligibility criteria for the trial. Pre-clinical disability was identified by asking the respondent;

1a For health or physical reasons do you have any difficulty in walking 800 meters?

1b Have you changed the way you walk 800 meters due to underlying health problems?

2a For health or physical reasons do you have any difficulty in climbing 10 steps?

2b Have you changed the way you climb 10 steps due to underlying health problems?

Participants who reported no difficulty with these mobility tasks were in the high function stage and therefore ineligible for the study; those who reported no difficulty but who have modified the task were in the pre-clinical stage of disability; and those who reported difficulty are advancing to manifest disability and were not eligible for the study[13]. The first 503 eligible participants, all of whom had been classified as pre-clinically disabled, were randomised to the larger study, of whom 471 completed all baseline performance and disability measurements and were subsequently included in this analysis. Participants were not eligible for randomisation into the larger study if: they were currently participating in Tai Chi, had moderate cognitive impairment as evidenced by an education or language adjusted score greater than 4 on the Short Portable Mental Status Questionnaire, had major unstable cardio-pulmonary disease, terminal cancer or other life threatening illness including a major psychiatric illness. All participants required approval from their family doctor. The study was approved by the Monash University Human Ethics Committee.

### Measurements

#### Demographic characteristics

Community dwelling participants were defined as those living independently in the community in a private dwelling, retirement dwelling participants lived in segregated congregate housing at the time of the study. Information on demographic characteristics including residential status was collected via a questionnaire sent to the participant prior to baseline physical assessment.

#### Health

Health status was measured in several ways. Self perceived health status (SPHS) on a 6 point scale (excellent, very good, good, fair, poor and very poor), self reported physician diagnosed chronic conditions and prescription medication use was collected via the questionnaire. Use of community health and support services in the previous four weeks (home help, home delivered meals, home nursing, community based allied health services, home maintenance, day care centre), hospital admissions in the previous four weeks, and falls in the previous twelve months were also collected via the questionnaire. The Beck Depression Inventory [17] was administered during the baseline performance measurement appointment, along with anthropometric measurements such as height and body mass index (BMI).

#### Physical performance

Objective instruments and tests to measure physical performance were completed during a 60 minute appointment. The 6-minute walk test measured cardiac function and mobility[18]. Muscle strength was assessed from the best of three attempts of the Spring Gauge test on bilateral quadriceps[19,20]. The timed chair stand test was used as a functional measure of general leg strength, the time taken to stand from a 45 cm high chair and sit down again three times was recorded. The timed chair stand was undertaken on a chair with no arms, participants were allowed to use their hands to push off from the chair or their thighs, if they were unable to do the chair stand otherwise. Postural sway, indicating static balance, was assessed using a sway meter that measures body displacement at waist level whilst standing barefoot with feet together eyes open and feet together eyes closed. Total sway path was recorded[21]. The length of time that single leg stance was maintained was also recorded as an indication of static balance. The timed up and go test (TUG-test) [22] measured mobility and dynamic balance, and the step test was performed to assess dynamic balance[23]. Both the TUG and step test have been well-validated[21-24]. Turning ability and bending were measured by two Berg Balance Scale assessment items (360 degree turn and picking up an item from the floor)[25]. Joint pain, stiffness and function were measured with the WOMAC Osteoarthritis Index[26].

#### Disability

Self reported physical functioning and disability were measured using the Late-Life Function and Disability Instrument (LLFDI). The LLDFI, conceptually founded on the Disablement Process and Nagi models, is one of the few disability instruments that provides a comprehensive assessment of all aspects of progressive disablement and disability. It has good concurrent and predictive validity and moderate to high test-retest reliability, while demonstrating no apparent floor or ceiling effects[27-29]. The function component evaluates difficulty in performing 32 physical activities in three dimensions: upper extremity, and basic and advanced lower extremity. The disability component evaluates limitations in, and frequency of, performing 16 major life tasks. The function and disability dimensions are all scored on a 0 to 100 scale.

### Statistical Analysis

Statistical analyses were conducted with STATA 10 (StataCorp, Texas, 2007) software as follows. First descriptive analyses were performed using number and proportion for all categorical variables while mean and standard deviation were used to describe continuous variables. Fulfilment of independence, homoscedasticity and of normality of model residuals in multiple linear regression models was examined. Chi-squared analysis was performed on all demographic and health variables to examine the difference between residential groups: retirement village dwelling and community dwelling older people. SPHS was collapsed to three categories: excellent and very good were categorised as very good; good remained as a category of its own; fair, poor and very poor were categorised as poor. Performance measures and self reported disability scores were examined comparatively using linear regression adjusting for potentially important factors identified as significantly different between residential groups: age, gender, living alone and education. The above comparative analyses were repeated to compare male and female participants.

To study factors that might lie on the theoretical pathway to progressive disablement, separate models were constructed for each of the three self reported disability components (function, frequency and limitation). Highly correlated explanatory variables were identified and were omitted from the models. These included: marital status, quadriceps strength, multiple falls, and knee and hip stiffness and function. Where bilateral measurements showed a high correlation, the right sided measurement was selected for inclusion in further analysis. In multivariable analysis the following factors, selected with reference to the Disablement Process model [9], were included to explore the variation of the disability components: gender, age, living alone, retirement village dwelling, education, number of chronic medical conditions, history of hypertension, stroke or arthritis, SPHS, number of falls, prescription medication use, 6 minute walk distance, height, BMI, right step test, postural sway with eyes closed, timed up and go, timed chair stands, single right leg stand, abbreviated Berg balance score, Beck depression score, knee pain score, hip pain score and the number of community services. Backwards stepwise estimation was performed using a significance level for removal from the model of p > 0.1.

## Results

### Demographic and health characteristics

Demographic characteristics for both residential groups are shown in Table 1. Over a fifth of our sample were retirement village dwellers (n = 105), with the greatest proportion of participants in the 75-79 year age group. In comparison, the greatest proportion of community dwellers was aged 70-74 years. Retirement village dwellers were more likely to be living alone (OR 2.4, CI 1.1-3.9, p < 0.001). There was a small difference in use of community services between residential groups, however there was no difference in health characteristics such as a history of chronic medical conditions known to affect functional decline including hypertension, stroke, and arthritis [30]. Consistent with other studies [18,27] a higher proportion of our sample were female (total sample population 68% female). There was no evidence that females differed from males by age, BMI, self perceived health status, the number of physician diagnosed chronic medical conditions, depression, falls or self reported prescription medication use (gender demographic data not shown). However, there was evidence that females were more likely to be living alone (OR 4.0, CI 2.6-6.5, p < 0.001), living in a retirement village (OR 1.8, CI 1.06-3.10, p = 0.02), have a history of arthritis (OR 2.0, CI 1.3-3.0, p < 0.001) and to have shorter stature (p < 0.001). Men were more likely to have a university education (OR 1.5, CI 1.03-2.4, p = 0.02).

**Table 1:** Comparison of demographic and health characteristics between community and retirement village dwellers, Exercise for Independent Living Study, Australia

Demographic and health characteristic	Community (n = 366)		Retirement (n = 105)		test	df	p
Gender*#	n	%	n	%			
Female	239	65.3	81	77.1	X^2 ^= 5.25	1	0.022
Age group*#							
70-74	134	36.6	22	21.0			
75-79	115	31.4	33	31.4			
80-84	82	22.4	30	28.6			
>85	35	9.6	20	19.1	X^2 ^= 13.63	3	0.003
**Marital status**#	.						
Married	182	50	41	39.4			
Single	15	4.1	7	6.7			
Widowed/separated/divorced	167	45.9	56	53.9	X^2 ^= 4.15	2	0.126
Lives alone*#							
Yes	154	42.7	67	64.4	X^2 ^= 15.34	1	0.000
Education*#							
Primary/some secondary school	119	32.8	49	47.1			
Secondary	72	19.8	20	19.2			
Trade	46	12.7	10	9.6			
College/university	126	34.7	25	24.0	X^2 ^= 8.11	3	0.044
**SPHS**#							
Very good	179	50.0	40	39.2			
Good	132	36.9	48	47.1			
Poor	47	13.1	14	13.7	X^2 ^= 4.06	2	0.131
**Hypertension**#							
Yes	197	54.6	54	51.4	X^2 ^= 0.32	1	0.500
**Stroke**#							
Yes	26	7.1	8	7.7	X^2 ^= 0.36	1	0.800
**Arthritis**#							
Yes	220	61.1	61	58.1	X^2 ^= 0.31	1	0.500
**History of falls in past 12 months**#							
Yes	111	30.3	28	26.7	X^2 ^= 0.61	1	0.400
**Hospital admissions in last month**							
Yes	18	5.1	3	3.1	X^2 ^= 0.7	1	0.400
**Prescription meds**#							
Yes	336	93.3	95	92.2	X^2 ^= 0.15	1	0.700
**Number of community services **mean(sd)*#	0.41	0.7	0.65	0.9	D = 0.144		0.051
**Height **mean(sd)*#	1.62	0.1	1.59	0.1	t = 2.820	469	0.005
**BMI **mean(sd) #	27.82	4.4	27.54	4.5	t = 0.564	467	0.570
**Number of medical conditions **mean(sd) #	2.30	1.4	2.24	1.4	t = 0.366	469	0.700
**Beck score **mean(sd) #	7.27	5.9	8.18	6.4	t = -1.373	469	0.170

*p < 0.05, #=*included in regression models, D = two-sample Kolmogorov-Smirnov test*

### Performance measures

Objective performance measures and reported disability status are shown in Tables 2 and 3. We use multivariable linear regression to adjust for factors known to affect functional performance including age, gender, and residential status [30,31], and those found to be different across residential settings: living alone and education level. There was some evidence for poorer postural sway with eyes closed among the retirement village dwellers (Table 2) and among men (Table 3), however the absolute difference in mean scores between the residential groups and between men and women was less than 0.30 mm and is therefore not likely to be clinically significant. There was moderate evidence for poorer balance and strength among the retirement village group, on the basis of the right and left single leg stand and left leg quadricep strength tests. Women had lower scores for the 6 minute walk test (p < 0.01), the timed up and go test (p = 0.033) and quadricep strength (right: p < 0.001, left; p < 0.001).

**Table 2:** Comparison of physical performance and function and disability scores between community and retirement village dwellers, Exercise for Independent Living study, Australia

Functional measures & Disability Status	Community (n = 366)		Retirement (n = 105)		Difference in mean	B	p
	*mean*	*SD*	*mean*	*SD*			
Timed up go (sec) #	9.4	2.8	10.1	2.8	0.7	0.31	0.299
Timed chair stands (sec) #	9.6	5.8	9.3	3.0	-0.3	-0.22	0.766
6 minute walk distance (m) #	403.1	95.1	392.8	87.8	-10.3	1.18	0.908
Postural sway open: total sway path (mm)	5.5	0.8	5.6	1.1	0.1	0.06	0.594
Postural sway closed: total sway path (mm) #	6.0	0.9	6.2	0.9	0.2	0.19	0.060
Berg balance score #	7.6	1.1	7.3	1.2	-0.2	-0.2	0.102
Step test right (steps) #	12.1	3.6	12.0	3.8	-0.2	0.002	0.996
Step test left (steps)	12.4	3.7	11.7	3.7	-0.7	-0.58	0.170
Quad strength right (kg)	20.4	8.8	17.3	6.8	-3.1	-1.06	0.192
Quad strength left (kg) *	20.7	9.0	17.0	7.3	-3.7	-1.63	0.051
Single right leg stand (sec) *#	16.5	17.9	9.6	14.2	-6.9	-4.58	0.022
Single left leg stand (sec) *	15.8	18.7	10.2	13.4	-5.7	-4.18	0.045
WOMAC:							
Knee pain score #	65.4	73.9	65.2	87.0	-0.2	-3.55	0.693
Knee stiffness score	34.5	41.5	32.0	39.7	-2.5	-4.69	0.329
Knee function score	283.9	266.9	286.2	271.2	2.3	-11.47	0.712
Hip pain score #	55.1	77.5	56.3	74.6	1.2	-1.95	0.827
Hip stiffness score	26.8	34.5	29.0	39.8	2.3	0.68	0.871
Hip function score	241.8	273.4	249.4	272.8	7.6	-5.84	0.855

LLFDI - Function total*	59.9	8.1	56.9	6.9	-3.0	-2.27	0.015
LLFDI - Disability freq	52.8	4.5	52.5	6.2	-0.3	0.14	0.810
LLFDI - Disability limitation	73.6	11.7	72.7	13.1	-0.9	-0.425	0.771

*p ≤ 0.05, #=*included in regression models*, B = *adjusted differences in means *

**Table 3:** Comparison of physical performance and functional and disability scores between male and female participants, Exercise for Independent Living study, Australia

Functional Measures and Disability Status	Males (n = 151)		Females (n = 320)		Difference in mean	B	p
	*mean*	*SD*	*mean*	*SD*			
Timed up go (sec)*	9.18	2.33	9.72	3.01	-0.54	0.60	0.033
Timed chair stands (sec)	9.19	4.09	9.32	3.43	-0.13	0.19	0.630
6 minute walk distance (m)*	416.74	96.22	393.22	91.43	23.52	-26.54	0.006
Postural sway closed: total sway path (mm)*	6.22	0.86	5.94	0.89	0.28	-0.31	0.002
Postural sway open: total sway path (mm)	5.58	0.83	5.54	0.92	0.04	-.060	0.538
Berg balance score	7.51	1.16	7.53	1.15	-0.02	.037	0.750
Step test right (steps)	12.30	3.46	12.00	3.72	0.30	-0.33	0.404
Step test left (steps)	12.31	3.62	12.20	3.74	0.11	-0.04	0.921
Quad strength right (kg)*	26.25	0.74	16.67	0.35	9.58	-9.40	<0.001
Quad strength left (kg) *	26.86	0.73	16.63	0.37	10.23	-10.22	<0.001
Single right leg stand (sec)	15.83	17.76	14.58	17.21	1.25	0.65	0.727
Single left leg stand (sec)	15.08	1.51	14.35	1.03	0.73	-0.47	0.809
WOMAC:							
Knee pain score1	56.95	67.44	69.35	80.89	-12.40	11.55	0.171
Knee stiffness score	29.29	36.52	36.07	42.98	-6.78	7.44	0.100
Knee function score	256.11	252.73	297.84	273.72	-41.73	45.31	0.125
Hip pain score1	50.31	67.04	57.78	80.92	-7.47	5.82	0.494
Hip stiffness score	24.18	30.91	28.74	37.77	-4.56	3.57	0.370
Hip function score	229.11	261.61	250.41	278.39	-21.3	16.72	0.582

LLFDI - Function total*	62.00	8.42	58.00	7.35	4.00	-4.19	<0.001
LLFDI - Disability freq*	51.94	4.41	53.14	5.06	-1.20	1.38	0.013
LLFDI - Disability limitation	73.55	11.72	73.33	12.12	0.22	-0.72	0.607

*p ≤ 0.05, B = *adjusted differences in means *

Participants residing in the community had on average a higher LLFDI functional dimension score by 3 scaled points (p = 0.015), and male participants had on average a higher functional dimension score by 4 scaled points (p < 0.001). Females had a slightly higher disability frequency score (p = 0.013). However the absolute differences in these mean scores are less than those that differentiate mild, moderate and severe functional categories in other studies [28], and may not be clinically meaningful. As expected, the function dimension of the LLFDI showed a positive correlation with the 6 minute walk test (r = 0.48, p < 0.001), the left step test (r = 0.38, p < 0.001) and bilateral quadricep strength (right; r = 0.37, p < 0.001 and left; r = 0.40, p < 0.001) and a negative correlation with the TUG (r = -0.47, p < 0.001) and timed chair stand test (r = -0.37, p < 0.001). The disability dimension scores for this sample population were not strongly correlated with objective performance measures, all correlations were less than 0.30 (data not shown).

### Models for pre-clinical disability

The results of the multiple linear regression models are shown in Table 4. Greater than fifty percent of the variation in the LLFDI function score was explained by the available factors (R^2 ^= 0.52). Consistent with theoretical models of progressive disablement [8,9] the statistical models identified a number of factors (risk, pathological, and impairment) and functional limitations strongly associated with function and disability. The well known risk factors of female gender and older age (80-84 and >85) were retained. Pathological factors were indicated by number of physician diagnosed medical conditions and history of arthritis. Associated impairments were indicated by knee and hip pain, height, BMI, SPHS and Beck depression score. Objective performance measures indicating functional limitations retained were the timed up go test and 6 minute walk. Retirement village dwelling and the number of community services used remained in the model. Factors associated with disability limitation (the extent to which people experienced limitation in life tasks) explained 35% of the variability in this outcome measure. The variance explained for the disability frequency outcome dimension was lower (R^2 ^= 0.20), suggesting there were other factors that explained the reported frequency of undertaking life tasks.

**Table 4:** Multivariate Linear Regression Models for Function and Disability (full sample n = 471),

Linear regression model	B	CI	p value
**Function total **R^2 ^= 0.515			
Female gender	-4.75	-6.73 to -2.77	<0.001
Age group 75-79	-1.37	-2.92 to 0.17	0.082
Age group 80-84	-1.88	-3.65 to -0.11	0.038
Age group >85	-2.46	-4.76 to -0.17	0.036
Number of community services	-0.82	-1.70 to 0.06	0.067
Retirement village dweller	-1.37	-2.87 to 0.13	0.074
Beck depression score	-0.23	-0.34 to -0.12	<0.001
Number of medical conditions	-0.50	-1.02 to 0.03	0.064
Timed up and go	-0.46	-0.76 to -0.16	0.003
Hip pain (WOMAC)	-0.01	-0.02 to 0.00	0.043
Arthritis	-1.61	-3.08 to -0.14	0.032
Knee pain (WOMAC)	-0.02	-0.03 to -0.01	<0.001
SPHS good	-0.18	-1.55 to 1.18	0.792
SPHS poor	-2.45	-4.74 to -0.17	0.035
BMI	-0.17	-0.33 to -0.02	0.031
6 minute walk distance (m)	0.01	0.01 to 0.02	0.002
Height	-11.04	-20.48 to -1.60	0.022
**Disability frequency **R^2 ^= 0.20			
Female gender	1.77	0.70 to 2.84	0.001
Beck depression score	-0.18	-0.26 to -0.09	<0.001
6 minute walk distance (m)	0.01	0.00 to 0.01	0.032
Step test (right leg stepping)	0.17	0.02 to 0.32	0.029
SPHS good	-1.00	-2.05 to 0.06	0.063
SPHS poor	-2.39	-4.20 to -0.59	0.010
Single leg stand (right)	0.03	0.00 to 0.06	0.028
Hypertension	0.91	-0.09 to 1.90	0.074
BMI	0.12	0.01 to 0.23	0.032
**Disability limitation **R^2 ^= 0.35			
BMI	0.29	0.04 to 0.53	0.024
SPHS good	0.66	-1.65 to 2.98	0.573
SPHS poor	-4.66	-8.74 to -0.57	0.026
Live alone	2.55	0.39 to 4.71	0.021
Number of community services	-1.74	-3.24 to -0.25	0.023
Secondary education	-1.81	-4.79 to 1.17	0.233
Trade education	1.80	-1.62 to 5.22	0.300
University education	-2.32	-4.88 to 0.24	0.075
Number of med conditions	-1.31	-2.14 to -0.48	0.002
Beck depression score	-0.42	-0.60 to -0.24	<0.001
Timed up and go	-0.71	-1.15 to -0.28	0.001
Knee pain (WOMAC)	-0.03	-0.04 to -0.01	0.002
Hip pain (WOMAC)	-0.02	-0.04 to 0.00	0.016

B = *adjusted differences in means, SPHS = self perceived health status, R^2 ^= adjusted R squared*

## Discussion

We found little evidence, among older people screened for pre-clinical disability, that retirement village dwellers were different to community dwellers in terms of their health characteristics, performance measures or self reported disability level. Previous studies in Australia [20] and America [31] have reported higher levels of functional impairment in retirement village dwellers than those living in the community, although residents in this form of accommodation in Australia are not usually expected to require assistance in personal care. These results suggest that using the Fried pre-clinical disability screening tool has facilitated the selection of a group of older Australian men and women from different residential settings with a comparable function and disability profile. While the retirement village dwellers in this study were more likely to be female, older, living alone, and using more community services, there was little difference between the groups in terms of their reported health status. The number of chronic medical conditions in both residential groups was comparable to that found in other groups of older community dwelling people with pre-clinical disability[13,15,32]. Unlike other studies we did not see a significant difference in depression between the residential settings [33] which is likely to be related to the similar physical health profile of our residential groups.

The retirement village dwellers performed slightly worse on some of the physical performance measures implying that there were underlying differences in the physical strength and balance characteristics of the two groups. However, we would argue that the group of people selected for the randomised study using the screening tool on average all have mild limitations in physical performance. There is little information on the expected TUG-test performance in retirement village dwellers. However, TUG-test performance differs between community dwelling and institutionalised older women[34]. Both groups in our study performed the TUG-test in ≤10 seconds consistent with other studies reporting scores for healthy community dwelling older people[24,35]. Quadriceps muscle strength (measured using a strain gauge) was significantly different for men and women, but showed only a small difference between residential settings. Measurements of muscle strength were 2 to 5 kg lower than those seen in studies of healthy community dwelling elders, although some differences may be due to different measurement apparatus and starting positions[19,24]. The single leg stand was the only performance test to show a significant difference between residential groups. The single leg stand test has been shown to have good sensitivity in discriminating age related differences [36] consistent with our findings among the comparatively older retirement village dwellers. While our single leg stand results showed a difference between groups, the fact that the mean retirement village score was comparable to healthy or mildly impaired older women in other studies implies that the retirement village group profile was still consistent with pre-clinical disability[12].

Despite a small number of differences in strength and balance, there was no clinically important difference in LLFDI function dimension scores between residential groups, and no difference in the disability frequency or limitation scores. This suggests that our sample population consists of older people with some functional limitation, but little perceived disability, consistent with the pre-clinical stage of mobility disability[7]. If we compare the mean LLFDI scores from our sample to those of other samples stratified according to level of physical function limitation, defined by the physical function items of the Short Form-36 Health Survey, the scores from our sample indicate a group with comparatively mild functional limitations (Table 5). LLFDI function scores for both male and female and community dwelling and retirement village dwellers indicated mild to moderate functional limitation[27,28]. Similarly, disability frequency and limitation scores for both male and female, and community dwelling and retirement village dwellers, were consistent with mild functional limitation[27,37].

**Table 5:** Comparison of Late Life Function and Disability Instrument (LLFDI) scores between this study and other reported studies.

Author	**Jette **[27]**, Haley**[28]			This Study				
**Study population**	Convenience sample of 150 USA community dwellers recruited by telephone, 60+ years, cognitively intact, able to get out of bed, no recent hospitalisation, stratified by physical function limitation defined by the physical function items of the SF-36.			AUST community and retirement dwellers, 70+ years, Pre-clinically disabled based on Fried screening tool.				

**Sample** **Size**	Severe limitation (n = 27)	Moderate limitation (n = 45)	Slight limitation (n = 57)	All (n = 471)	Community (n = 366)	Retirement (n = 105)	Men (n = 151)	Women (n = 320)

**LLFDI dimension:**	*mean(SE)*							
Function Total	41.7 (7.0)	53.2 (6.2)	65.6 (6.9)	59.3 (7.9)	59.9 (8.1)	56.9 (6.9)	62 (8.4)	58 (7.4)
Disability frequency	44.3 (4.9)	49.5 (4.9)	53.6 (4.8)	52.8 (4.9)	52.8 (4.5)	52.5 (6.2)	51.9 (4.4)	53.1 (5.1)
Disability limitation	55.4 (7.3)	63.5 (8.3)	73.8 (10.9)	73.4 (12)	73.6 (11.7)	72.7 (13.1)	73.6 (11.7)	73.3 (12.1)

SE = standard error

Our statistical models identified a range of factors associated with function, disability frequency and disability limitation, which explained a greater degree of the variation in function, than disability. All the models showed that, in addition to random variation, there were likely to be factors, other than those measured in this study, which contributed significantly to the variation in function and disability. Female gender was a major factor associated with function and disability in our models consistent with longitudinal studies that suggest that gender differences in disability prevalence are related to the prevalence of chronic conditions [38] and the higher incidence and longer duration of disability [39] among older women. Increasing age, a well known and significant risk factor for disability [40], was particularly applicable to the function component of our statistical model. Performance in the 6 minute walk and TUG-test indicated the physical measures most strongly associated with functional impairment, as might be expected from existing research suggesting that lower extremity function strongly predicts subsequent disability[14]. We found a lower BMI to be associated with functional limitation and suggest that this measure represents a marker of advancing frailty, which has been described as a physiologic precursor and etiologic factor in disability, due to its central features of weakness, decreased endurance, and slowed performance[41].

Consistent with previous studies [30,40], we identified the contribution of joint complaints to levels of function and disability. Interventions to address joint complaints are likely to contribute to higher levels of physical activity in older people which has been shown to be protective against the development of disability [42]. The importance of self perceived health status and depression in characterising mobility disability is also highlighted in our models, and well supported in the literature[14]. The results are consistent with these factors being on the pathway to progressive disablement and mobility limitation and add weight to existing theoretical models that predict impairment factors associated with early functional loss[8,9]. These findings offer insight into the type of impairment factors that could be addressed through disability prevention policy relevant to both community dwelling or retirement village dwelling older men and women. Of some interest was the observation that the models appeared to explain early functional decline rather than overt disability. This might imply that the onset of disability may be heralded by a major event such as the onset of a new illness on the background of loss of physiological reserve.

A limitation of this study was the cross sectional design which did not allow us to examine the predictive validity of the screening tool as a means of identifying older people at risk of progressing to manifest disability. Further research is required to confirm studies from other populations where predictive validity has been examined[13,15]. We were unable to determine the sensitivity of the screening tool to detect people with pre-clinical disability as information on participants that were not eligible for participation was not available. Nonetheless the screening tool has been shown to have good test-retest reliability in determining pre-clinical mobility disability in community dwelling older Americans[13,16]. We were unable to explore the difference in function and disability in retirement village and community dwelling older people in general. It would be expected that a greater difference in function and disability would be seen in a less selected, random, sample. As participants in this study were volunteers and were required to meet strict inclusion criteria, the generalisability of the statistical modeling results is somewhat limited. The strength of this study lies in the large number of male and female participants that were able to be selected from different residential settings using the screening tool.

## Conclusion

Our findings corroborate risk factors for functional limitation and disability previously published by others. In addition, the Fried pre-clinical disability tool identified a group of people with a function and disability profile reflecting early functional limitation consistent with the pre-clinical stage of disability. While these results demonstrate the application of a screening tool to identify a homogenous study or intervention sample from different populations, this information also adds to the knowledge concerning the use of the screening tool and allows for greater confidence in its use for research, clinical, and population health purposes. Furthermore the ease and low cost of administration allow for its use in public health policy aimed at preventing disability and offering effective interventions to vulnerable older people during early decline in functional status.

## Abbreviations

BMI: Body Mass Index; LLFDI: Late Life Function and Disability Index; SPHS: Self Perceived Health Status; TUG: Timed Up and Go test; OR: Odds Ratio; CI: Confidence Interval.

## Competing interests

The authors declare that they have no competing interests.

## Authors' contributions

KG and LD conceived the idea for this paper. KG undertook the analysis, guided by SN and DJ, and drafted the manuscript. LD, FC, LS, KDH, LF and DJ made substantial contributions to the conception of the larger RCT, in which this study is nested. All authors contributed to interpretation of the results, provided critical comment on the manuscript, and approved the final version.

## Pre-publication history

The pre-publication history for this paper can be accessed here:

http://www.biomedcentral.com/1471-2318/10/52/prepub
